# The influence of afferent input on somatosensory suppression during grasping

**DOI:** 10.1038/s41598-020-75610-8

**Published:** 2020-10-29

**Authors:** Maximilian Davide Broda, Katja Fiehler, Dimitris Voudouris

**Affiliations:** 1 Experimental Psychology, Justus Liebig University, Giessen, Germany; 2 Center for Mind, Brain and Behavior (CMBB), University of Marburg and Justus Liebig University, Giessen, Germany

**Keywords:** Human behaviour, Motor control, Sensorimotor processing, Sensory processing, Somatosensory system

## Abstract

The processing of somatosensory information is hampered on a moving limb. This suppression has been widely attributed to sensorimotor predictions that suppress the associated feedback, though postdictive mechanisms are also involved. Here, we investigated the extent to which suppression on a limb is influenced by backward somatosensory signals, such as afferents associated with forces that this limb applies. Participants grasped and lifted objects of symmetric and asymmetric mass distributions using a precision grip. We probed somatosensory processing at the moment of the grasp by presenting a vibrotactile stimulus either on the thumb or index finger and asked participants to report if they felt this stimulus. Participants applied greater forces with the thumb and index finger for objects loaded to the thumb’s or index finger’s endpoint, respectively. However, suppression was not influenced by the different applied forces. Suppression on the digits remained constant both when grasping heavier objects, and thus applying even greater forces, and when probing suppression on the skin over the muscle that controlled force application. These results support the idea that somatosensory suppression is predictive in nature while backward masking may only play a minor role in somatosensory processing on the moving hand, at least in this context.

## Introduction

Somatosensory signals are typically attenuated when presented on a limb that is about to move^1,2^ or is moving^3–5^ compared to when that limb is at rest. This phenomenon of somatosensory suppression has been attributed to sensorimotor predictions established by a forward model that estimates future sensory states by integrating sensory feedback with an efference copy of the motor command^6^. When the predicted and actual sensory outcomes match, associated afferences are suppressed^7–9^. Interestingly, external somatosensory signals that are not predicted by efference copies can also be attenuated on a moving limb, suggesting a general reduction of somatosensory sensitivity^2,4^. The strength of suppression depends on the relative utilization of predictive and afferent signals^9,10^: When the sensorimotor prediction is more reliable, for instance when the underlying dynamics are fully predictable, afferent signals from the moving limb are down-weighted and somatosensory suppression is greater^10^.

Somatosensory suppression has also been explained by backward masking mechanisms^11–13^. For instance, externally generated stimuli on a limb are suppressed even when that limb is only passively moved. During passive movements sensorimotor predictions are unlikely to occur, as there is no expected motor command and, thus, no efference copy. Because processing somatosensory stimuli on a stimulated limb takes time, subsequent signals arising from that limb can influence the perception of the previously presented stimuli^12,14^, explaining why suppression is evident even before the onset of a passive movement^12^. Such suppression appears to be stronger on moving digits of the hand than on more proximal parts of the moving forearm^12^, possibly because the density of mechanoreceptors responsible for sensory processing is greater on the digits^15^, and this may lead to greater transmission of backward signals during digit movements than movements of more proximal joints.

The strength of backward masking has been shown to depend on the intensity of the afferent signals, with stronger suppression in the presence of stronger backward signals^16^. Suppression is also stronger when the externally generated stimulus is in close temporal proximity to the backward signals^14,17^. We also recently observed that the strength of suppression on a grasping hand may be related to the strength of afferent sensory information from that hand^10^. More specifically, suppression on the grasping index finger appeared more pronounced when this finger was more likely to apply greater forces when grasping to lift an asymmetrically loaded object^10^, in which case different digits apply different amount of forces to reassure efficient object lift and manipulation^18^. Greater force production is associated with greater muscle activations^19^, and greater muscle activations can lead to stronger transmission of afferent information^20^. For example, Golgi tendon organs that mainly encode actively produced force^21^ discharge more vigorously when the muscle is actively contracting^20^. If the strength of somatosensory suppression is sensitive to the transmission of afferent signals, then suppression on a limb should depend on the amount of the forces applied by this limb.

To examine this, we asked participants to reach, grasp and lift an object that could have one of three possible mass distributions: left, central and right. Importantly, participants were to place their digits on the left and right sides of the object. By requiring participants to lift this object as straight as possible and by restricting their digits’ placement on the object, we could implicitly manipulate the amount of forces that each digit had to exert in order to contribute to the required lift off. To evaluate somatosensory processing on the grasping digits, we presented a probing vibrotactile stimulus either on the thumb or the index finger at the moment of that digit’s contact with the object. We expected greater object roll to the left and right for the respective asymmetric mass distributions. We also expected stronger forces for the thumb and index finger when grasping objects loaded on the side of the thumb’s and index finger’s endpoint^18^. We further expected somatosensory suppression during grasping compared to rest^10^. Importantly, if the strength of suppression depends on backward masking mechanisms, suppression should be stronger with increased afferent signals, so we expect stronger suppression on digits that apply stronger forces during object lift-off.

## Methods Experiment 1

### Participants

Fourteen participants completed the experiment. Due to the exclusion criteria mentioned below (see “Data analysis” section), our final sample included 12 participants (7 women, 5 men; range: 19–32 years; *M* = 24.33; *SD* = 3.34), who were all right-handed according to the German translation of the Edinburgh Handedness Inventory^22^ (78.35 ± 6.26). All participants provided informed written consent and received 8€/hour or course credits for participating in the experiment. The experiment was approved by the ethics committee at the Justus Liebig University Giessen and was in accordance with the Declaration of Helsinki (2008).

### Apparatus

**Figure 1:**
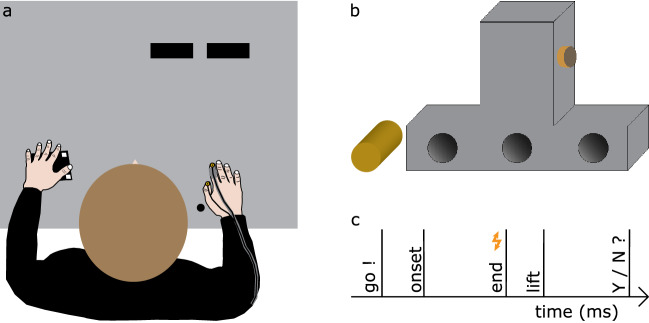
Experimental setup and timeline. (**a**) Top view of the setup with participants sitting in front of a table. The start position is indicated by the black circle. Participants reached to grasp and lift an object placed at one of two possible target positions (black rectangles). (**b**) Participant’s view of the object that could have one of three possible mass distributions by having a piece of aluminium in one of the three slots at its lower compartment. Participants were to grasp the object with the thumb and index finger on the left and right side of the object’s upper part. The digits could only be placed at the force sensors, one of which is depicted as orange circle with a touch sensor placed on top (dark grey). (**c**) Timeline of a grasping trial. A go-cue prompted participants to start reaching to grasp and lift the object. At the moment of object contact, a probing vibrotactile stimulus was presented on the grasping thumb or index finger and participants were to respond whether and, if yes, where they detected the stimulus.

Participants sat comfortably in front of a table (118 × 80 cm) having their right shoulder aligned with a start position on the table, ~ 30 cm from their body. To avoid stereotypical grasping movements, we placed the target object at one of two target positions: Each of these target positions was 34 cm away from the start position and they were 15 cm apart from each other in the lateral direction. A schematic top-view of the setup is depicted in Fig. 1.

Participants reached to grasp a custom-made object that had an inverted T-shape. It was made of polyoxymethylene and consisted of an upper (2 × 7 × 6 cm) and a lower part (15 × 4 × 6 cm). Three tubes (diameter: 3 cm; depth: 5 cm) were distributed symmetrically along its lateral axis of the object’s lower part. A piece of aluminium (100 g) was fit in one of these tubes, so that the object could have either one symmetric or two asymmetric mass distributions. The object was always placed in such a way that participants could see where the piece of aluminum was fit, so participants knew its mass distribution.

Two force sensors (diameter: 1.7 cm; height: 1.4 cm; ATI Industrial Automation, Apex, NC) were attached on the same height of the object’s upper part, one on each side. These sensors were connected through a NI USB-6225 device (National Instruments Corporation, Austin, TX) to the host PC and recorded the three-dimensional forces and torques that the participant applied when grasping the object (1000 Hz). Each force sensor was covered with an additional touch sensor (diameter: 1.8 cm; Interlink Electronics Inc., Westlake Village, CA), each of which was connected through a NI USB-6009 device (National Instruments Corporation, Austin, TX) to the host PC and used to determine the moment of object contact online during the experiment. Considering all attached equipment, the resulting object’s mass was ~ 300 g.

A custom-made vibrotactile stimulation device (Engineering Acoustics, Inc., Casselberry, FL) was used to present probing vibrotactile stimuli (250 Hz, 25 ms) of seven possible amplitudes. These stimuli were presented by two small tactors, each of which was attached to the proximal phalanx of the dorsal part of the participants’ right thumb and index finger. The stimuli on each digit were presented at the moment of object contact of that digit, determined by the touch sensors (see above). The seven stimulation amplitudes ranged from 0 (no stimulus) to 56.7 µm in steps of 9.4 µm.

Lastly, the position of two infrared markers on the participants’ right thumb and index finger nails and of another three markers on the backside of the object (with respect to the participant) were recorded at 100 Hz with an Optotrak Certus motion tracking system (Northern Digital, Inc., Waterloo, ON, Canada).

### Procedure

Participants performed four blocks of trials: one baseline block without movement and three grasping blocks. We used the baseline block to assess somatosensory sensitivity on each participant’s thumb and index finger when no movement was performed. To this end, participants were asked to only detect the presence of a probing stimulus on one of their digits. In each of the grasping blocks, participants reached to grasp and lift the object and then reported whether and where they detected a tactile stimulus. We applied the same probing stimuli in the grasping and the baseline blocks. The mass distributions were held constant in the three grasping blocks.

In the baseline block, participants placed their right hand comfortably in front of them with the palm facing down. A probing stimulus was presented on their thumb or index finger at the start of each trial. After the presentation of an auditory cue, participants reported whether they had felt a stimulus or not by pressing a key with their left hand. In case of a negative answer, the trial was over. In case of a positive answer, a second auditory cue was presented prompting participants to report which digit was stimulated by pressing one of two foot-pedals. The next trial started 500 ms after the participants’ last response.

Before each grasping block, participants performed eight practice trials, all with the same mass distribution that would be presented during the upcoming block. Participants placed their right hand on the start position with their thumb and index finger touching each other. The experimenter placed the object at the target position and pressed a button to start the trial. An auditory go-cue notified participants that they could start their reach-to-grasp movement. Their task was to grasp the object at the force sensors with only their thumb and index finger and lift it as straight as possible ~ 10 cm high. Participants were encouraged to grasp and lift the object at their own speed within a time frame of 4000 ms after the go-cue. Probing stimuli on the thumb or index finger could be presented only at the moment of contact of the probed digit, as this moment was determined online by the respective touch sensor. After placing the object back to the target position, participants returned their hand to the start position. Similar to the baseline block, an auditory cue then prompted participants to respond whether they had felt a stimulus during the trial by pressing a button with their left hand. In case of a negative answer, the trial was over. If they responded that they had felt a stimulus, another auditory cue informed participants to indicate by a foot-pedal press which digit was stimulated. After their last response, the next trial started. The target position was randomized within each block.

Half of the participants started the experiment with the baseline block while the other half ended the experiment with the baseline block. The order of presentation of the grasping blocks was randomized and counterbalanced across participants and the various stimulus amplitudes were presented in pseudo-randomized order within each block. For both the baseline and the grasping blocks, each of the 6 physical stimulus amplitudes was repeated 6 times and the no-stimulation catch trials were repeated 12 times on each digit. This resulted in a total of 96 trials for both digits in each (baseline and grasping) block. The baseline block lasted ~ 4 min and each grasping block took ~ 13 min.

### Data analysis

For our offline kinematic analyses, we first calculated the three-dimensional speed of the hand by numerical differentiation of the average position of the two markers on the participants’ thumb and index finger. Movement onset was determined on the basis of the first of 5 consecutive frames with a three-dimensional speed criterion of > 20 cm/s. Similarly, the onset of the object’s lift was determined on the basis of the first of 5 consecutive frames in which the object’s vertical velocity was > 20 cm/s. We computed movement end as the moment between movement onset and object lift onset when the speed of the hand was minimal. This represents the moment of contact with the object for our offline analyses.

To assess the influence of different mass distributions on object lift, we calculated *maximal object roll* as the maximal angle between the horizontal plane and the object based on the object markers. Object roll was calculated from the onset of the object’s lift until 250 ms later^10,18^, with positive and negative values indicating rolling to the participants’ right and left, respectively.

Our main interest was to examine whether the strength of somatosensory processing on the moving hand is influenced by increased afferent signals caused by greater applied digit forces. To this end, we first examined whether the forces that each digit applied were modulated across the three mass distributions. We calculated two force measures separately for the thumb and index finger, namely *maximal grip force* and *maximal load force*. Grip force was defined as the force applied orthogonally to the force sensors and load force as the force normal to that surface, i.e. a vertical lift force. Maximal grip and load forces were determined within a time frame between force sensor contact until 500 ms later, a time frame that is thought to include maximal grasping forces^23^. Force sensor contact was defined separately for each grasping force of both digits as the moment in which the according force was 6 standard deviations higher than the according baseline force, which was defined as the average force during the first 200 ms of each trial.

Subsequently we examined whether changes in somatosensory processing on the grasping digits were associated with the applied forces by the digit in question. To this end, we first fit a psychometric function to the participants’ responses to the probing stimuli. For each participant, we fit separate psychometric functions for each digit and mass distribution using the maximum-likelihood estimation of the *psignifit* toolbox^24^ in MATLAB. We only considered trials in which participants correctly reported the presence of stimulus on the correct digit. We then calculated the detection threshold as the 50% point of the psychometric function. To examine whether somatosensory processing was hampered during grasping compared to rest, we subtracted the baseline detection threshold of each participant’s digit from their respective threshold during each grasping block. Thus, for each participant we obtained 6 values (3 mass distributions, 2 digits), termed threshold_diff_, which represent the strength of somatosensory suppression during grasping, with greater positive values indicating greater suppression.

Participants with a detection threshold beyond the stimulus range in any of the grasping blocks were excluded from further data analyses. Furthermore, we excluded individual trials from the maximal object roll analysis if object lift onset was later than 3750 ms after the start of the trial, because this meant that there were not enough samples to measure object roll during the first 250 ms after object lift (89 trials, 2.6% of total trials). In addition, we excluded trials from the force analysis if any object contact was later than 3500 ms after the start of the trial (because we looked for maximal forces during the first 500 ms after object contact), if no force sensor contact was detected, or if the baseline force was higher than 0.01 N (in total 9 trials, < 1% of total trials).

To establish the expected effects of mass distribution (left, center, right) on kinematic behavior^10^, we calculated a repeated-measures ANOVA for maximal object roll. To confirm that somatosensory processing was hampered during movement compared to rest^4,25^, we tested whether each of the 6 calculated threshold_diff_ values was significantly different from zero by using one-sided *t*-tests.

To examine whether the applied forces as well as somatosensory processing were modulated across mass distributions separately for each digit, we calculated a 3 × 2 repeated-measures ANOVAs separately for maximal grip force, maximal load force and threshold_diff_. For all above-mentioned ANOVAs, when sphericity was violated, Greenhouse–Geisser correction was applied. Significant interactions revealed by the ANOVAs were further investigated with post-hoc one-way ANOVAs. Significant main effects were further explored with post-hoc two-sided paired *t*-tests. All *t*-tests were Bonferroni-Holm corrected.

## Results Experiment 1

### Kinematic results

**Figure 2:**
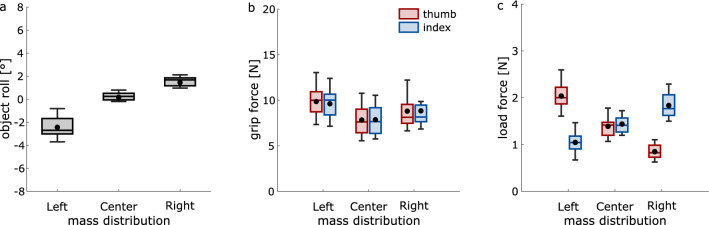
Behavioral results. Effects of mass distribution on (**a**) maximal object roll, (**b**) maximal grip force, and (**c**) maximal load force. Negative and positive values in (**a**) show object rolls to the left and right, respectively. Box plots show averages and medians across participants along with the lower and upper quartiles and whiskers (± 1.5 * interquartile range).

Maximal object roll during lift off was significantly affected by the mass distribution (*F*_1.084, 11.926_ = 82.36, *p* < 0.001, η_p_^2^ = 0.88; Fig. 2a): Objects with left and right mass distributions leaned further to the left and right during lift, respectively (all *t* > 8.28, all *p* < 0.001). This is not surprising because the designated contact points with the object did not allow for adjustments of digit placement along the object’s vertical axis, thereby leading to clear object roll^10,18^.

For maximal grip force, there was a main effect of mass distribution (*F*_2, 22_ = 12.17, *p* < 0.001, η_p_^2^ = 0.53; Fig. 2b), showing greater grip forces for left compared to central mass distributions (*t*_11_ = 5.19, *p* < 0.001). No other comparison was significant (all *t* < 2.56, all *p* > 0.026). There was no main effect of digit (*F*_1, 11_ = 3.00, *p* = 0.111, η_p_^2^ = 0.21) or interaction between mass distribution and digit (*F*_1.374, 15.112_ = 2.67, *p* = 0.115, η_p_^2^ = 0.20).

For maximal load force, there was a main effect of mass distribution (*F*_2, 22_ = 11.76, *p* < 0.001, η_p_^2^ = 0.52; Fig. 2c), with greater load forces for left than right (*t*_11_ = 3.94, *p* = 0.002) and central distributions (*t*_11_ = 3.72, *p* = 0.003), but not between right and central distributions (*t*_11_ = − 1.89, *p* = 0.086). There was no main effect of digit (*F*_1, 11_ = 0.08, *p* = 0.777, η_p_^2^ = 0.01). Our main interest, though, was whether the amount of applied forces was differently modulated across distributions for each digit. This was indeed the case, as we found a significant interaction between mass distribution and digit (*F*_2, 22_ = 467.35, *p* < 0.001, η_p_^2^ = 0.98). Separate ANOVAs for each digit revealed a main effect of mass distribution for both the thumb (*F*_1.231, 13.537_ = 317.79, *p* < 0.001, η_p_^2^ = 0.97) and index finger (*F*_2, 22_ = 94.52, *p* < 0.001, η_p_^2^ = 0.90), with maximal load force increasing from right to left distributions for the thumb (all *t* > 13.6, all *p* < 0.001) and from left to right distributions for the index finger (all *t* > 6.78, all *p* < 0.001). In short, participants applied greater forces with the digit that was placed at the loaded side of the object, confirming our hypothesis.

### Somatosensory processing

**Figure 3:**
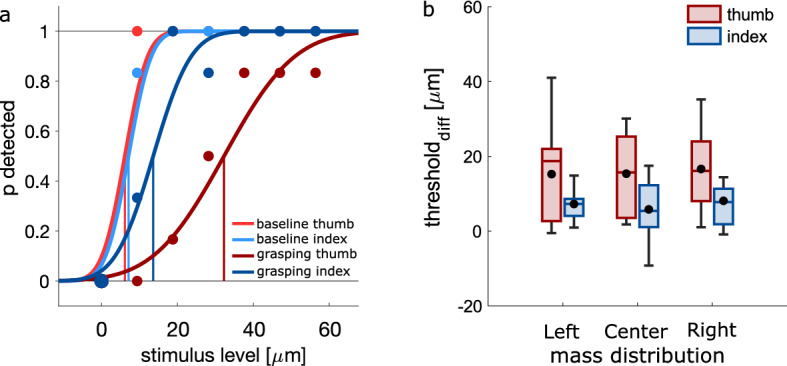
Somatosensory processing. (**a**) Representative psychometric functions of a single participant indicating somatosensory processing on each digit during baseline and grasping. (**b**) Strength of somatosensory suppression during grasping for each digit and each mass distribution. Greater values represent stronger suppression. Details as in Fig. 2.

Somatosensory processing was hampered when grasping compared to rest, as expected^4,10^. This was evident for both stimulated digits for the left (thumb: *t*_11_ = 4.20, *p* < 0.001; index: *t*_11_ = 5.99, *p* < 0.001), central (thumb: *t*_11_ = 4.99, *p* < 0.001; index: *t*_11_ = 2.71, *p* = 0.010) and right (thumb: *t*_11_ = 5.33, *p* < 0.001; index: *t*_11_ = 3.73, *p* = 0.002) mass distribution (Fig. 3).

Our main question, though, was whether suppression would be greater in the presence of stronger afferent signals. If this would be the case, we expected stronger suppression in conditions when the applied forces would be greater, resulting in an interaction between mass distribution and digit, similarly to what we found for maximal load force. This, however, was not the case (*F*_2, 22_ = 0.15, *p* = 0.866, η_p_^2^ = 0.01). To further explore whether suppression was related to the amount of applied forces, we correlated the strength of suppression of both digits separately with maximal grip and load forces for each individual participant, but found no systematic relationship (both *r* < 0.116, both *p* > 0.336). These results suggest that the hampered somatosensory processing on the thumb and index fingers during grasping was not modulated by the amount of applied forces. Lastly, there was no main effect of mass distribution (*F*_2, 22_ = 0.74, *p* = 0.489, η_p_^2^ = 0.06), but, interestingly, suppression on the thumb was greater than the index finger (*F*_1, 11_ = 7.22, *p* = 0.021, η_p_^2^ = 0.40; Fig. 3).

## Discussion Experiment 1

In Experiment 1 we examined whether the strength of somatosensory suppression is influenced by stronger afferent input. To this end, we asked participants to grasp and lift an object that had one of three different mass distributions along the final grip axis, so we implicitly required participants to apply different forces with each digit when grasping and lifting the object. We indeed found that, for each digit, maximal load force was influenced by the mass distribution, with the thumb and index finger applying greater forces when grasping objects loaded to the thumb’s and index finger’s endpoint, respectively. Although somatosensory processing on both digits was hampered when grasping compared to rest, as expected^1,4,10^, there was no interaction between the object’s mass distribution and the probed digit, nor any correlation between the strength of suppression and the strength of the applied forces. Therefore, the strength of afferent information from the grasping digits seems to have no influence on somatosensory processing on these digits.

Interestingly, we found that suppression was systematically stronger on the thumb than the index finger, independently of the object’s mass distribution. This cannot result from the amount of forces applied by the thumb because there was no difference in the grip or load force between the digits. To explore this unexpected finding, we conducted a second experiment, in which we increased the mass of the target object. We expected an increase in maximal grasping forces based on previous findings^26^. If the greater suppression on the thumb compared to the index finger is a stable effect, we expect to find a similar relationship as in Experiment 1. In addition, with this manipulation we could further examine whether a modulatory effect of backward signals on the strength of suppression is elicited only when these signals are greater.

## Methods Experiment 2

Nineteen participants completed this experiment. Due to the exclusion criteria mentioned above (see Methods of Experiment 1), our final sample included 12 participants (8 women, 4 men; range: 19–26 years; *M* = 22.25; *SD* = 2.45), who were all right-handed according to the German translation of the Edinburgh Handedness Inventory^22^ (91.29 ± 3.34). None of them had participated in Experiment 1. All participants provided informed written consent and received 8€/hour or course credits for participating in the experiment. The experiment was approved by the ethics committee at the Justus Liebig University Giessen and was in accordance with the Declaration of Helsinki (2008). The apparatus, procedure and data analysis were identical to those of Experiment 1, except for the details mentioned below.

In Experiment 2, we exchanged the piece of aluminium with a heavier piece of brass (diameter: 3 cm; depth: 5 cm; weight: 192 g) to examine possible effects of greater applied forces on suppression. To confirm that the heavier object influenced grasping behavior in Experiment 2, we first calculated for each participant their *absolute* maximal object roll, maximal load force and maximal grip force for each of the three mass distributions and we then averaged across these three values. By doing so for each of the two Experiments, we could examine between-subject differences with separate *t*-tests for the *absolute maximal object roll, absolute grasping forces* and *absolute threshold*_*diff*_. We excluded 37 trials (1.1% of all trials) from the maximal object roll analysis and 35 trials (1% of all trials) from the force analysis.

## Results Experiment 2

### Kinematic results

Using a heavier object in Experiment 2 led to greater absolute maximal object roll (*t*_22_ = − 4.79, *p* < 0.001) and greater grasping forces (maximal grip force: *t*_22_ = − 1.69, *p* = 0.106; maximal load force: *t*_22_ = − 3.70, *p* = 0.001) as compared to Experiment 1.

**Figure 4:**
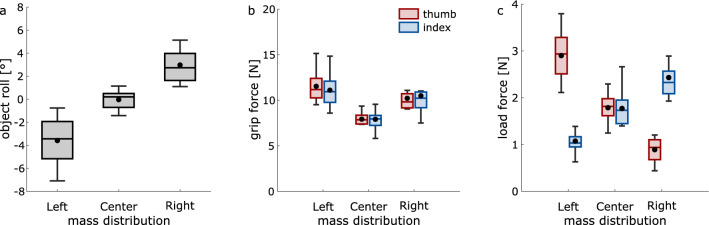
Behavioral results in Experiment 2. Effects of mass distribution on (**a**) maximal object roll, (**b**) maximal grip force, and (**c**) maximal load force. Details as in Fig. 2.

As expected, the mass distribution influenced the maximal object roll (*F*_2, 22_ = 123.69, *p* < 0.001, η_p_^2^ = 0.92; Fig. 4a), as roll was greater toward the left and right for distributions to the left and right compared to the central (all *t* > 8.45, all *p* < 0.001).

Maximal grip force was influenced by the mass distribution (*F*_2, 22_ = 24.29, *p* < 0.001, η_p_^2^ = 0.69; Fig. 4b), as it was stronger for left (*t*_11_ = 7.01, *p* < 0.001) and right (*t*_11_ = 4.51, *p* = 0.001) distributions compared to the central. There was no main effect of digit (*F*_1, 11_ = 1.49, *p* = 0.248, η_p_^2^ = 0.12), but an interaction between mass distribution and digit (*F*_2, 22_ = 12.96, *p* < 0.001, η_p_^2^ = 0.54), caused by effects of mass distribution on both the thumb (*F*_2, 22_ = 26.23, *p* < 0.001, η_p_^2^ = 0.71) and index finger (*F*_2, 22_ = 21.99, *p* < 0.001, η_p_^2^ = 0.67): Both digits applied greater forces in the left (thumb: *t*_11_ = 7.39, *p* < 0.001; index: *t*_11_ = 6.53, *p* < 0.001) and right (thumb: *t*_11_ = 4.34, *p* = 0.001; index: *t*_11_ = 4.61, *p* = 0.001) than central distribution. Maximal grip force was slightly greater in the left than right distribution for the thumb (*t*_11_ = 2.66, *p* = 0.022), but not for the index finger (*t*_11_ = 1.28, *p* = 0.225).

Maximal load force was again influenced by the mass distribution (*F*_2, 22_ = 23.49, *p* < 0.001, η_p_^2^ = 0.68; Fig. 4c), with greater forces for left than central (*t*_11_ = 3.83, *p* = 0.003) and right distributions (*t*_11_ = 6.15, *p* < 0.001), and lower forces for right than central distributions (*t*_11_ = − 3.41, *p* = 0.006). Importantly, we again found an interaction between mass distribution and digit (*F*_1.337, 14.708_ = 344.39, *p* < 0.001, η_p_^2^ = 0.97): The mass distribution influenced differently the load forces of the thumb (*F*_2, 22_ = 298.69, *p* < 0.001, η_p_^2^ = 0.96) and of the index finger (*F*_2, 22_ = 151.46, *p* < 0.001, η_p_^2^ = 0.93), with greater and lower thumb forces for left and right distributions, respectively (all *t* > 13.3, all *p* < 0.001), and greater and lower index finger forces for right and left distributions, respectively (all *t* > 9.6, all *p* < 0.001).

### Somatosensory processing

**Figure 5:**
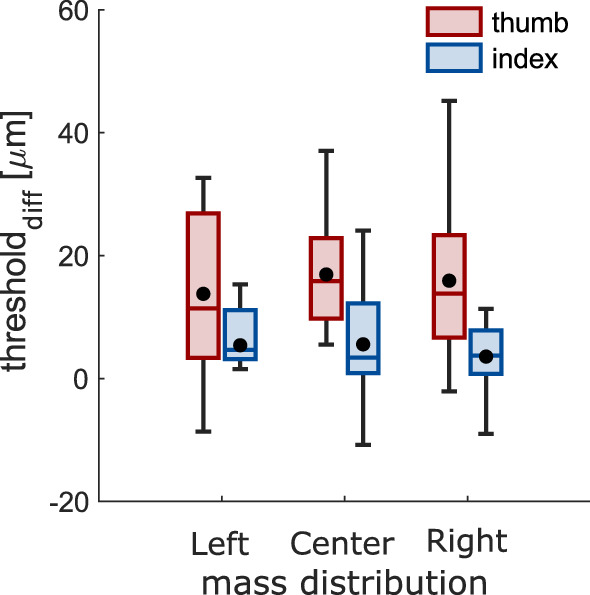
Somatosensory processing in Experiment 2. Strength of somatosensory suppression during grasping for each digit and each mass distribution. Details as in Fig. 3.

Somatosensory processing was again hampered while grasping compared to rest (all *t*_11_ > 1.97, all *p* < 0.037; Fig. 5). There was no main effect of mass distribution (*F*_2, 22_ = 0.53, *p* = 0.597, η_p_^2^ = 0.05). There was also no interaction (*F*_1.358, 14.941_ = 0.56, *p* = 0.578, η_p_^2^ = 0.05). The strength of suppression was similar to Experiment 1 (*t*_*22*_ = 0.415, *p* = 0.682). There was again no correlation between the strength of suppression and the maximal grip or load force (both *r* < 0.460, both *p* > 0.088). Importantly, there was a main effect of digit (*F*_1, 11_ = 23.59, *p* = 0.001, η_p_^2^ = 0.68), again with stronger suppression on the thumb than the index finger.

## Discussion Experiment 2

In Experiment 2 we explored possible reasons for the stronger suppression on the thumb than the index finger that we found in Experiment 1. We examined whether this difference would preserve when the required forces to grasp and lift the object would be greater. In addition, by employing a heavier object we further investigated whether suppression would increase compared to Experiment 1, which would provide evidence for a relationship between afferent input and the strength of suppression. As hypothesized, the heavier object led to an increase in maximal grasping forces. However, the overall suppression was similar between the two experiments, but, interestingly, suppression was again greater on the thumb than on the index finger. Why was this again the case?

The increased suppression on the thumb cannot be a result of greater backward masking, as we did not find any relationship between the applied forces and the strength of suppression neither in Experiment 1 nor in Experiment 2. However, because the applied forces are not generated at the probing sites (digit phalanges), but rather by the muscles controlling the digits, it might be that examining somatosensory processing on a body part farther away from its controlling muscle attenuates the strength of the associated suppression. For instance, suppression is weaker when probed at body parts further away from the moving limb^25^. This might explain why suppression was stronger on the thumb than the index finger in Experiments 1 and 2, as the probing site of the thumb was closer to its parent muscle than that of the index finger. To resolve this, we conducted a third experiment, in which we presented the probing stimulus on the skin just over muscles that control thumb and index finger force application. If the strength of suppression differed between the two digits in Experiment 1 and 2 because of predictive or postdictive mechanisms that influence differently somatosensory processing on the thumb and index finger, then suppression should again be greater on the thumb than the index finger. Alternatively, if the previously observed suppression arose due to different distances of the probing stimuli from the thumb’s and index finger’s parent muscles, then suppression should now be similar when probed at the skin over these muscles.

## Methods Experiment 3

Seventeen participants completed this experiment. Due to the exclusion criteria mentioned above (see Methods of Experiment 1) and due to the recording problems with one additional participant, our final sample included 12 participants (7 women, 5 men; range: 19–30 years; *M* = 24.17; *SD* = 4.09), who were all right-handed according to the German translation of the Edinburgh Handedness Inventory^22^ (81.11 ± 3.69). None of them took part in Experiment 1 or 2. All participants provided informed written consent and received 8€/hour or course credits for participating in the experiment. The experiment was approved by the ethics committee at the Justus Liebig University Giessen and was in accordance with the Declaration of Helsinki (2008). The apparatus, procedure and data analysis were identical to those of Experiment 1, except that we now presented the probing stimulus on the muscles controlling the thumb (thenar eminence) and index finger (first dorsal interosseus hand muscle). Following the previously mentioned criteria, we excluded 108 trials from the maximal object roll analysis (3.1% of all trials) and 40 trials from the force analysis (1.2% of all trials).

## Results Experiment 3

### Kinematic results

**Figure 6:**
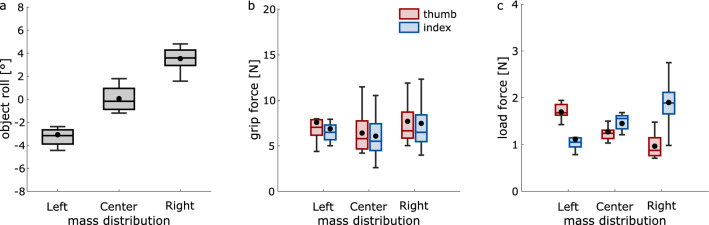
Behavioral results in Experiment 3. Effects of mass distribution on (**a**) maximal object roll, (**b**) maximal grip force, and (**c**) maximal load force. Details as in Fig. 2.

Maximal object roll was again influenced by the mass distribution (*F*_2, 22_ = 127.32, *p* < 0.001, η_p_^2^ = 0.92; Fig. 6a), as the object rolled more to the side of the external mass (all *t* > 8.68, all *p* < 0.001).

Maximal grip force was influenced by the mass distribution (*F*_2, 22_ = 7.87, *p* = 0.003, η_p_^2^ = 0.42; Fig. 6b) with forces being greater for left (*t*_11_ = 2.86, *p* = 0.016) and right (*t*_11_ = 3.76, *p* = 0.003) compared to central distributions. There was no effect of digit (*F*_1, 11_ = 3.55, *p* = 0.086, η_p_^2^ = 0.24). An interaction between mass distribution and digit (*F*_2, 22_ = 7.09, *p* = 0.004, η_p_^2^ = 0.39) stemmed from effects of mass distribution on both the thumb (*F*_2, 22_ = 8.77, *p* = 0.002, η_p_^2^ = 0.44) and index finger (*F*_2, 22_ = 7.08, *p* = 0.004, η_p_^2^ = 0.39), with greater forces of the thumb for left vs. central distributions (thumb: *t*_11_ = 3.62, *p* = 0.004) and of both digits for right vs. central distributions (thumb: *t*_11_ = 3.73, *p* = 0.003; index: *t*_11_ = 3.69, *p* = 0.004).

Maximal load force was not influenced by the mass distribution (*F*_2, 22_ = 0.71, *p* = 0.502, η_p_^2^ = 0.06; Fig. 6c), but it was greater on the index finger than the thumb (*F*_1, 11_ = 7.16, *p* = 0.022, η_p_^2^ = 0.39). Importantly, we again found an interaction between mass distribution and digit (*F*_1.123, 12.353_ = 95.91, *p* < 0.001, η_p_^2^ = 0.90), stemming from different influences of the mass distribution on the forces of each digit (thumb: *F*_2, 22_ = 33.97, *p* < 0.001, η_p_^2^ = 0.76; index finger: *F*_2, 22_ = 57.79, *p* < 0.001, η_p_^2^ = 0.84): As the distributions shifted further to the left and further to the right, maximal load forces were greater for the thumb (all t > 6.42, all p < 0.005) and index finger (all t > 5.7, all p < 0.005), respectively.

### Somatosensory processing

**Figure 7:**
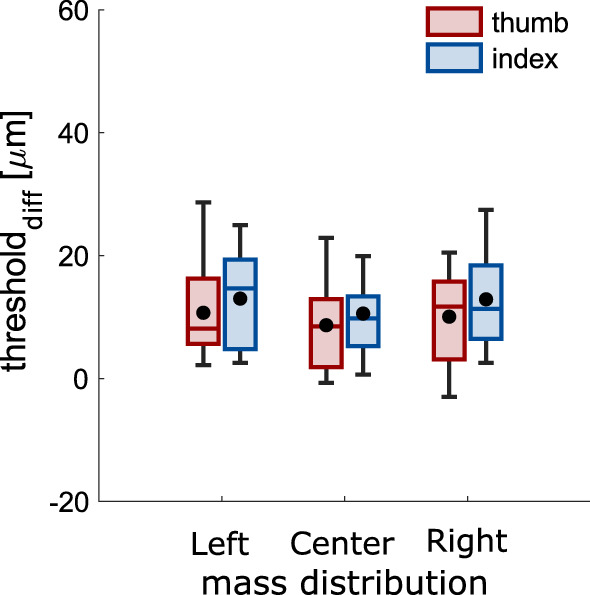
Somatosensory processing in Experiment 3. Strength of somatosensory suppression during grasping for each digit and each mass distribution. Details as in Fig. 3.

Somatosensory processing was hampered during grasping compared to rest in both stimulated muscles and for all mass distributions (all *t* > 4.11, all *p* < 0.002; Fig. 7). There was again no effect of mass distribution (*F*_2, 22_ = 1.58, *p* = 0.229, η_p_^2^ = 0.13), nor any interaction between mass distribution and digit (*F*_2, 22_ = 0.04, *p* = 0.959, η_p_^2^ < 0.01). A correlation between the strength of suppression and maximal grip force (*r* = − 0.388, *p* = 0.001) was in the opposite direction than what one might expect if suppression would increase with stronger afferent input. Maximal load force did not correlate with the strength of suppression (both *r* = 0.020, both *p* = 0.869). Importantly, the persistent difference in suppression between thumb and index finger that we found in the previous two experiments was now absent (*F*_1, 11_ = 1.28, *p* = 0.281, η_p_^2^ = 0.10).

## Discussion Experiment 3

In Experiment 3 we examined whether the differences in suppression between thumb and index finger that we found in Experiments 1 and 2 were due to differences in the distance between the probing area on each digit and the parent muscle that controlled the force application of that digit. Instead of probing somatosensory processing on the proximal phalanges of the thumb and index finger as before, we now measured somatosensory processing on the skin just over the muscles controlling the force application of these digits. This way, the probing site of each digit was equidistant from their respective control muscles. This eliminated the previously observed differences in suppression between the digits. In Experiment 3, suppression on the index finger increased while suppression on the thumb remained the same, which support the idea that differences in suppression between thumb and index finger in Experiments 1 and 2 were likely due to the different distances between the probing sites and the muscles controlling the force application of each digit. This is in line with previous findings showing weaker suppression when probed at body parts that are further away from the origin of the movement^25^. Similar to the previous two experiments, we again did not find any connection between the strength of suppression and the strength of the applied forces.

## General discussion

Somatosensory suppression has mainly been explained by predictive mechanisms that hamper somatosensory sensitivity on a moving limb by down-weighting somatosensory afferences^9,10,27^. Nevertheless, postdictive mechanisms also play a role through backward sensory signals that mask the perception of probing stimuli applied on the limb to measure somatosensory sensitivity^11,12^. In this study, we examined the extent to which the strength of somatosensory suppression on a limb depends on the strength of backward signals from that limb. To this end, we investigated whether suppression on the thumb and index finger is stronger with stronger forces that these digits apply when grasping to lift an asymmetrically distributed object. We found no evidence for a modulating effect of backward signals on somatosensory suppression.

Grasping to lift asymmetrically loaded objects requires the adoption of a strategically suitable grip orientation^10,28^ and predictive as well as reactive force application strategies^26^ to counteract the dynamics of the object. By constraining digit placement on the object, the effects of the asymmetric mass distribution can only be reduced by tailoring one’s forces when grasping to lift the object^18^. As we also constrained digit placement, our participants did apply greater load forces with their thumb and the index finger when grasping objects loaded to the side of the thumb’s and index finger’s endpoint, respectively. Because counteracting object roll of asymmetrically distributed objects requires both the adjustment of contact points and the modulation of the grasping forces^18^, object roll in our experiments was influenced by the mass distribution, despite the tailored load forces of the thumb and index finger.

By having participants apply different levels of grasping forces with their digits, we could examine whether the strength of somatosensory suppression on these digits depends on the strength of the applied forces. This was based on findings that show greater rates of backward sensory signals with greater muscles activations^20^ and greater suppression on the digits than the forearm^12^, possibly due to the greater amount of mechanoreceptors on the digits and, therefore, the afferent information they convey^15^. Contrary to our hypothesis, though, there was no indication that the strength of suppression is modulated by backward masking, as this would be caused by stronger forces. In addition, even though we increased the object’s mass between Experiments 1 and 2, we did not observe any increase in suppression. Similarly, we did not find any indication for masking effects when stimulating the muscles controlling the force application of the respective digits (Experiment 3), and there were no correlations between the strength of suppression and the amount of applied forces in any of the three experiments. These results suggest that although backward masking mechanisms may be involved in causing somatosensory suppression, their role in modulating the strength of this suppression is rather minor. Moreover, these results strengthen the idea that suppression is shaped mainly by sensorimotor predictions^2,10,29^.

It might be possible that backward signals did not modulate the strength of suppression because afferent information associated with generating and maintaining adequate grasping forces was relevant for controlling the task^30,31^. In this case, the need to process sensory feedback signals from the grasping digits might compromise any suppression of afferent information from these digits. Indeed, suppression, and somatosensory processing in general, can be modulated by the relevance of the available sensory signals to the task at hand^1,3,13,17,32,33^. Therefore, if afferent input from the grasping digits in our study was important for controlling the grasp-to-lift action, somatosensory processing on these digits may not be hampered. Although this would reflect a dynamic tuning of the backward sensory stream on suppression, this may be rather unlikely because a core idea of suppression from backward masking is that masking effects are greater with stronger reafferent input^16^.

An unexpected result that we found in Experiment 1 and 2 was that somatosensory suppression was more pronounced on the thumb than on the index finger. In addition, when looking at individual data, it was mostly the hampered somatosensory processing on the thumb that led to the exclusion of participants in Experiments 1 and 2. Because we did not observe differences in suppression between the digits in Experiment 3, the stronger suppression on the thumb might be related to a stronger sensorimotor prediction about the thumb’s sensory state. This could be in line with notions that humans control their thumb when grasping, based on findings that the thumb moves along a less variable trajectory than the index finger^34–36^, although such postulations have been challenged^37,38^. Our results of Experiment 3 rather suggest that the differences in suppression between thumb and index finger in Experiments 1 and 2 arose because of differences in the proximity between the probing sites and the parent muscle of each digit. For example, probing stimuli presented at body parts further away from the moving limb are suppressed less^11^, so probing suppression on the skin over the controlling muscle of each digit, eliminated the differences between the digits. Although previous studies have examined suppression on different digits of a grasping hand^22,39^, to the best of our knowledge, none of the studies so far had directly compared somatosensory suppression on the thumb and index finger, digits that have a key role in grasping.

In sum, in the three presented experiments we show that the strength of somatosensory suppression on a grasping hand is unlikely to be modulated by the strength of backward signals. This questions the versatility of the notion that suppression stems from backward masking mechanisms^11,12^ and suggests that, although postdictive mechanisms play some role in suppression, these mechanisms do not appear to have a modulatory effect. Our results strengthen the notion that somatosensory suppression is mainly driven by predictive mechanisms^9,10^.

## Data Availability

The datasets supporting the presented results are available at: OSF.IO/CT5M8.
